# Biocompatible coated magnetosome minerals with various organization and cellular interaction properties induce cytotoxicity towards RG-2 and GL-261 glioma cells in the presence of an alternating magnetic field

**DOI:** 10.1186/s12951-017-0293-2

**Published:** 2017-10-17

**Authors:** Yasmina Hamdous, Imène Chebbi, Chalani Mandawala, Raphael Le Fèvre, François Guyot, Olivier Seksek, Edouard Alphandéry

**Affiliations:** 1 Nanobacterie, 36 boulevard Flandrin, 75116 Paris, France; 2 Laboratoire d’Imagerie et Modélisation en Neurobiologie et Cancérologie (IMNC), Campus Universitaire, Bât. 440, 15 rue Georges Clemenceau, 91406 Orsay Cedex, France; 3 Institut de Minéralogie de Physique des Matériaux et de Cosmochimie, UMR 7590, CNRS, Université Pierre et Marie Curie, Muséum National d’Histoire Naturelle, Sorbonne Université, 4 Place Jussieu, 75005 Paris, France; 4 Institut de Physique du Globe de Paris, Sorbonne Paris Cité, UMR 7154, CNRS, Université Paris Diderot, 1 rue Jussieu, 75005 Paris, France

**Keywords:** Magnetosomes, Magnetotactic bacteria, Magnetosome minerals, Magnetic hyperthermia, Alternating magnetic field, Cancer, Oncology, Nanomedicine, Iron oxide, nanoparticle, nanotechnology, nanooncology

## Abstract

**Background:**

Biologics magnetics nanoparticles, magnetosomes, attract attention because of their magnetic characteristics and potential applications. The aim of the present study was to develop and characterize novel magnetosomes, which were extracted from magnetotactic bacteria, purified to produce apyrogen magnetosome minerals, and then coated with Chitosan, Neridronate, or Polyethyleneimine. It yielded stable magnetosomes designated as M-Chi, M-Neri, and M-PEI, respectively. Nanoparticle biocompatibility was evaluated on mouse fibroblast cells (3T3), mouse glioblastoma cells (GL-261) and rat glioblastoma cells (RG-2). We also tested these nanoparticles for magnetic hyperthermia treatment of tumor in vitro on two tumor cell lines GL-261 and RG-2 under the application of an alternating magnetic field. Heating, efficacy and internalization properties were then evaluated.

**Results:**

Nanoparticles coated with chitosan, polyethyleneimine and neridronate are apyrogen, biocompatible and stable in aqueous suspension. The presence of a thin coating in M-Chi and M-PEI favors an arrangement in chains of the magnetosomes, similar to that observed in magnetosomes directly extracted from magnetotactic bacteria, while the thick matrix embedding M-Neri leads to structures with an average thickness of 3.5 µm^2^ per magnetosome mineral. In the presence of GL-261 cells and upon the application of an alternating magnetic field, M-PEI and M-Chi lead to the highest specific absorption rates of 120–125 W/g_Fe_. Furthermore, while M-Chi lead to rather low rates of cellular internalization, M-PEI strongly associate to cells, a property modulated by the application of an alternating magnetic field.

**Conclusions:**

Coating of purified magnetosome minerals can therefore be chosen to control the interactions of nanoparticles with cells, organization of the minerals, as well as heating and cytotoxicity properties, which are important parameters to be considered in the design of a magnetic hyperthermia treatment of tumor.

**Electronic supplementary material:**

The online version of this article (doi:10.1186/s12951-017-0293-2) contains supplementary material, which is available to authorized users.

## Background

Magnetic nanoparticles have been developed in nanomedicine [1, 2], as improved magnetic resonance imaging contrast agents eéc, as nano-heater in the magnetic hyperthermia treatment of cancer [3], or as efficient nano-carriers used for targeted drug delivery [4]. To reach optimal conditions, these applications usually require high magnetization, uniform morphology, narrow size distribution and good dispersion in desired solvents, a series of properties that can’t easily be achieved with chemical synthesis. Interestingly, a certain type of bacterium, called magnetotactic bacterium, MTB, naturally synthesizes magnetic iron oxide nanocrystals, called magnetosomes that are used by these bacteria as a compass to navigate in the direction of the earth magnetic field to find the optimum environment for their growth and survival. Magnetosomes, which are made of a ferrimagnetic mineral iron oxide core, enveloped by a biological membrane essentially containing phospholipids and proteins [5], fulfill all the requirements mentioned above with regard to size, morphology, biocompatibility and magnetization properties [6]. Under the application of an alternating magnetic field, AMF, magnetosomes produce at the same concentrations larger temperature increases than most chemically synthesized magnetic nanoparticles [7]. Magnetosome therefore seem particularly well suited for the treatment of tumor using magnetic hyperthermia [8–10] and for other applications such as diagnosis [11]. Their industrial development has been fostered by new high yield magnetosome production methods [12]. However, MTB, which are Gram-negative bacteria, contain lipo-polysaccharides or bacterial endotoxins [13], that may cause fever or septic shock. Their presence is therefore prohibited in a medicinal product [14].

In this study, to obtain a suspension containing sterile and endotoxin-free magnetosomes, most biological material surrounding the magnetosome mineral core was therefore removed. Following bacterial lysis, chains of magnetosome were obtained, which underwent a purification process to yield nonpyrogenic uncoated magnetosome minerals. The latter were prone to random interactions due to their high magnetization, resulting in magnetosome aggregation and sedimentation in aqueous suspension [15]. To obtain a stable suspension, uncoated magnetosome minerals were therefore coated with three different substances commonly used in medical products: (i) chitosan [poly(1,4-β-d-glucosamine)], which is a biodegradable natural polysaccharide polymer [16], previously used to coat iron oxide nanoparticles [17], (ii) neridronate, which is a bisphosphonate molecule with two phosphonic acid groups that may bind to divalent or trivalent metals [18], used for the treatment of osteoporosis and Paget disease under the name of Nerixia^®^ [19], (iii) polyethyleneimine (PEI), which is a cationic polymer with several primary amino groups [20], used as an in vitro and in vivo transfection agent known as RNAi jetPEI [21], previously used to stabilize iron oxide [22].

Furthermore, heating, cytotoxic and cellular internalization properties of M-Chi, M-Neri, and M-PEI, were examined and compared with those of uncoated magnetosome minerals and pyrogenic chains of magnetosomes directly extracted from magnetotactic bacteria. Cytotoxicity was studied both on healthy 3T3 cells and on tumor GL-261 and RG-2 glioblastoma cells. To study antitumor efficacy of these different magnetosomes for potential use in the magnetic hyperthermia treatment of tumors, M-Chi, M-Neri, and M-PEI, were brought into contact with GL-261 and RG-2 glioblastoma tumor cells and exposed to an AMF of 198 kHz and strength of 34–47 mT that produces temperatures of 43–46 °C, which are required for efficient magnetic hyperthermia treatment of tumors. To assess in vitro efficacy, the following parameters were measured following AMF application: the decrease in the percentage of living GL-261 and RG-2 cells, the quantity of magnetosomes internalized/externalized in/from cells and the magnetosome specific absorption rate (SAR).

## Methods

### Culture of magnetotactic bacteria

*Magnetospirillum gryphiswaldense* strain MSR-1 (DSMZ 6361) is purchased from Deutsche Sammlung von Mikro-organismen und Zellkulturen (Brunswick, Germany) and stored at −80 °C. An aliquot of 1 mL is thawed, 40 µL of cell suspension are deposited and grown at 29 °C on solid agar medium under microaerobic conditions for 7 days [23]. Several colonies are then collected from the solid agar medium, cultivated and amplified at 29 °C under stirring at 120 rpm in a growth medium devoid of iron [24]. Cells are then diluted in a 35 L fermentation medium and fermentation is carried out at 29–30 °C under agitation at 200 rpm during 5 days at a pH, which is maintained at 6.9 by adding an acidic medium containing an iron source [24]. Growth of magnetotactic bacteria is stimulated by bubbling oxygen in the growth medium. During growth, foaming is suppressed by adding 200 µL per liter of polypropylene glycol of feeding medium. Temperature, agitation speed, pH, feeding pump flow and oxygen concentration are monitored and adjusted using an EZ-controller and a BioXpert software from Applikon Biotechnology.

### Preparation of suspensions containing chains of magnetosomes extracted from magnetotactic bacteria, MC

Following fermentation, MSR-1 cells are first concentrated and washed in water using tangential flow filtration (mPES KrosFlo^®^ Filter Modules Part No. K02-E20U-05-N). To lyse the bacteria, concentrated MSR-1 cells are re-suspended in 5 M NaOH and heated at 80 °C during 2 h in a sonicating bath (SB) at 25 kHz. After bacterial lysis, magnetosomes are separated from the organic material using a Neodymium magnet overnight. Magnetosomes are then washed 4 times using 1× Phosphate-buffered saline (PBS). During each wash, the magnetosome suspension is sonicated at 10 W for 20 s (Digital Branson Sonifier, head model 1020) and is then positioned against a Neodymium magnet during 20 min that attracts the magnetosomes. The supernate containing organic debris is then removed and replaced by water. At the end, suspensions containing magnetosome chains surrounded by a biological membrane are thus obtained and autoclaved.

### Preparation of uncoated magnetosome minerals, M-uncoated

Suspensions containing chains of magnetosome, MC, are re-suspended in five different solutions: firstly in 1× PBS under sonication at 10 W using the sonicating finger (SF) during 20 s, secondly in 1% Triton X-100 and 1% SDS under heating at 60 °C overnight, thirdly in phenol at pH 8 under heating at 60 °C during 2 h in the sonicating bath (SB), fourthly in chloroform under heating at 60 °C during 2 h, fifthly in 1 M NaOH under heating at 60 °C during 1 h in the SB. Following each of the different treatments with detergents, magnetosome minerals are magnetically isolated from organic debris using a Neodymium magnet for 20 min that attracts the magnetosome minerals. The supernate of the suspension containing magnetosome minerals and organic debris is then removed and replaced by the next detergent or non-pyrogenic water after the fifth step. Suspensions containing uncoated magnetosome minerals, M-uncoated, with a low percentage of organic materials coming from the bacteria are thus obtained. They are autoclaved and stored at −80 °C.

### Preparation of coated magnetosome minerals, M-PEI, M-Chi, M-Neri

In order to produce the different suspensions of coated magnetosome minerals, three different solutions are first prepared containing: (i) 160 mg of a chitosan powder (Molar mass (MM): 600–800 kDa, degree of deacetylation (DD): 90% from Acros Organics) dissolved in 8 mL of a 1% (w/v) acetic acid solution mixed using mechanical stirring until the solution is transparent and adjusted to pH 4.0 using 0.1 M NaOH, (ii) 200 mg of PEI (MM: 60 kDa from Acros Organics) dissolved in 8 mL of water, (iii) 160 mg of a Neridronate powder (MM: 277.15 Da from Sigma Aldrich) dissolved in 8 mL of water. Each solution is filtered with a polyethersulfone filter of 0.2 µm for sterilization. 1 mL of a suspension of uncoated magnetosome minerals at 20 mg/mL in iron is then exposed to a strong magnetic field for 5 min using a Neodymium magnet. The supernate of the suspension of uncoated magnetosome minerals is removed and replaced by 8 mL of each of the three solutions containing chitosan, PEI or Neridronate. The different assemblages are then sonicated either in the SB during 5 h at room temperature for preparing M-Chi or using a SF at 30 W during 1 h 30 for synthetizing M-PEI or M-Neri. Coated magnetosome minerals are then collected through magnetic separation and washed three times using nonpyrogenic water.

### Characterization

#### Transmission electron microscopy (TEM)

TEM is carried out using a *JEOL JEM2100* operated at 200 kV. The samples studied by TEM contain 7 µL of each nanoparticle suspension at 300 µg/mL in iron deposited on top of a carbon-coated copper grid. The suspensions are left to dry during one night before carrying out TEM observations. Particle sizes and size distributions are estimated using 440 particles.

#### ξ-potential measurement

Dynamic light scattering measurements are carried out on the different suspensions of nanoparticles in water using *Zetasizer Nano ZS Malvern* Instrument. The ξ-potential of each sample, which is diluted at 30 µg/mL in iron, is measured at pH varied from 2 to 11 by introducing NaOH or HCl at 25 °C. For reproducibility, at least three measurements are carried out at each pH value.

#### Colloidal stability

Variations with time of the absorption of the different suspensions of nanoparticles are measured using an *UviLine9400 Secomam* spectrophotometer. For that, 1 mg in iron of each magnetosome suspension dispersed in 1 mL of water, and medium cell culture (DMEM 10% FBS and RPMI 20% FBS) are introduced in a quartz cuvette after rapid homogenization and the variation of the absorption of the suspension is measured at 476 nm during 20 min. The experiments are carried out in triplicate.

#### Fourier transform infrared (FTIR)

FTIR measurements are carried out on a lyophilized powder of nanoparticles. FTIR spectra are recorded using the transmission mode (*Brucker Vertex 70*) using the attenuated total reflection (ATR Pike Germanium) spectra technology (scanning range 4000–400 cm^−1^, resolution of 4 cm^−1^, number of scans: 400).

#### CHNS elemental analyzer

CHNS measurements are carried out on a CHNS analyzer (*Analyzer Elemental Flash EA 1112 from Thermo Fischer Scientific*) using 3 mg in iron of lyophilized nanoparticle suspension to determine the percentage in weight of carbon, nitrogen, hydrogen and sulfur in each preparation. The experiments are carried out in triplicate.

#### Concentration in iron of the different magnetosome suspensions

Total iron concentration of the different nanoparticle suspensions are determined by a spectrophotometric method. Magnetosome minerals are digested in HCl 12 N during 2 h at 70 °C; the solution is then mixed with hydrogen peroxide to produce Fe^3+^ ions complexed with potassium thiocyanate, whose quantity is determined by absorbance at 476 nm using *UviLine 9400 Secomam* spectrophotometer.

#### LAL assay

The detection and quantification of bacterial endotoxins are carried out with a LAL assay, under sterile conditions using the 88282 ThermoScientific kit called *‘‘Pierce LAL Chromogenic Endotoxin Quantitation Kit*’’ 0.1 mL of each nanoparticle suspension at a concentration of 100 µg in iron per mL is homogenized by sonication, and heated at 70 °C for 10 min required to denature any residual proteins that could interfere with the LAL assay. 10 µL of each suspension containing 1 µg in iron are then introduced in a well with 15 µL of Endotoxin-free water and maintained at a temperature of 37 °C throughout the duration of the experiment. 25 µL of the LAL solution kit are added to initiate the reaction. After 10 min of reaction, 50 µL of the chromogenic substrate are added to the well for 6 min and the amount of endotoxins is detected. Finally, 25 µL of acetic acid are added to stop the reaction. The optical density of the obtained suspensions is measured at 405 nm using a microplate reader. The endotoxin concentration is then estimated for 1 µg of nanoparticles in iron using a calibrating curve plotted according to the kit instructions and expressed as endotoxin unit (EU) per mL per mg of iron contained in nanoparticles (Table 1). In other words, Table 1 reports endotoxin concentrations for magnetosome suspensions of concentration 1 mg/mL. To avoid that the presence of nanoparticles interferes with the results, we use a rather low quantity of nanoparticles of 1 µg in iron in the assay.

**Table 1:** Endotoxin concentration, estimated in endotoxin unit, EU, per mg in iron per ml of Mg, M-uncoated, M-PEI, M-Chi, M-Neri, measured with the LAL assay

	Endotoxin concentration per iron (EU/mL/mg Fe)
MC	2000–12,000
M-uncoated	18–130
M-PEI	12–124
M-Chi	157–194
M-Neri	35–203

### In vitro studies

#### Growth of 3T3, RG-2 and GL-261 cells

The mouse fibroblast healthy 3T3 cell line is purchased from American Type Culture Collection (ATCC^®^CCL-163™) and maintained at 37 °C in a 5% (v/v) CO_2_ incubator in Dulbecco’s modified Eagle’s medium (DMEM) supplemented with 5% (v/v) Newborn calf serum (NBCS) and with 20 mM of 4-(2-hydroxyethyl)-1-piperazineethanesulfonic acid (HEPES). Rat glioblastoma RG-2 cells are purchased from ATCC^®^ (*CRL*-*2433™*) and maintained in DMEM (*SH 30081.01 from HyClone*) supplemented with 10% fetal bovine serum (FBS), l-glutamine (0.584 g/L) (*25030*-*024* *from Gibco*), sodium pyruvate (0.11 g/L) (*11360*-*039 from HyClone*), penicillin G sodium (50 units/ml) and streptomycin sulfate (50 μg/mL) (*SV30010 from HyClone*) at 37 °C in a humidified incubator containing 5% CO_2_. Mouse glioblastoma GL-261 cells are purchased from NCI Tumor Repository, Frederickand, maintained in Roswell Park Memorial Institute medium (RPMI 1640) with l-glutamine (*SH30027.01 from HyClone*) supplemented with 20% FBS at 37 °C in a humidified incubator containing 5% CO_2_. The different cells are grown in 175 cm^2^ culture flasks. Confluent cell monolayers are trypsinized, cells are harvested during the exponentially growing phase and used for cytotoxicity experiments.

#### Neutral red uptake, NRU, assay on healthy 3T3 cells

Cytotoxicity of magnetosome chains, M-uncoated, M-Chi, M-Neri, and M-PEI, are measured on healthy 3T3 cells using a NRU assay, according to ISO 10993-5 standard. This technique measures the accumulation of neutral red dye in the lysosomes of living cells. 3T3 cells are seeded into 96-well plates (10^4^ cells per well) and maintained in culture for 24 h at 37 °C and 5% CO_2_. After incubation, the medium culture is removed and replaced by 100 µL of culture medium containing suspension of magnetosome chains (MC), M-uncoated, M-Chi, M-Neri, and M-PEI, at different iron concentrations of 16, 31, 62, 125, 250, 500, or 1000 µg/mL. Cells are incubated at 37 °C in a 5% CO_2_ humidified atmosphere for 24 h. Following incubation, the culture medium is removed and cells are washed once with 150 µL of a PBS solution. Cells are then incubated with 100 µL of Neutral red medium at 50 µg/mL for 3 h at 37 °C in 5% CO_2_. After that, cells are washed once with 150 µL of a PBS solution and 150 µL of a Neutral red desorbing fixative (EtOH/AcCOOH, 50%/1%) are added to the cells, followed by cell shaking for 10 min to lead to complete dissolution of neutral red. Using a microplate spectrophotometer system, optical densities at 540 nm of cells alone, OD_UC_, or of cells mixed with the different magnetosomes and treated as described above, OD_TC_, are measured. The percentage of cell inhibition, % inhibition, is then calculated using the formula: % inhibition = [1 − (OD_TC_/OD_UC_)] × 100, where OD_UC_ is the optical density, measured at 540 nm, of the suspension of untreated cell, and OD_TC_ is the optical density, measured at 540 nm, of the suspension of treated cells. The experiments are carried out in triplicates. Dose–response curves are plotted for the different suspensions. These experiments were carried out in triplicate.

#### MTT assay on RG-2 and GL-261 tumor cell lines

Cytotoxicity of MC, M-uncoated, M-Chi, M-Neri, and M-PEI, is estimated using an MTT assay, carried out on GL-261 and RG-2 tumor cell lines. This technique measures the ability of mitochondrial enzymes to reduce 3-(4,5-dimethylthiazol-2-yl)-2,5-diphenyltetrazolium bromide to purple formazan crystals. GL-261 and RG-2 cells are seeded with 100 µL of culture medium in individual wells of a plate containing 96 wells (10^4^ cells per well) and allowed to adhere overnight. Then, the culture medium is removed and replaced by 100 µL of culture medium containing magnetosome chains (MC), M-uncoated, M-Chi, M-Neri, and M-PEI at different iron concentrations of 16, 31, 62, 125, 250, 500, or 1000 µg/mL, and the cells are incubated at 37 °C in a 5% CO_2_ humidified atmosphere during 24 or 72 h. Following incubation, cells are washed with a PBS solution and incubated with 100 µL of MTT at 1 mg/mL for an additional 3 h at 37 °C. The insoluble product is then dissolved by addition of 100 µL of an isopropanol solution. The absorbance of solubilized formazan, estimated at 540 nm using a microplate reader, measures the percentage of inhibition of living cells. The percentage of cell inhibition, inhibition (%), is then calculated using the formula: % inhibition = [1 − (OD_TC_/OD_UC_)] × 100, where OD_UC_ is the optical density, measured at 540 nm, of the suspension of untreated cell, and OD_TC_ is the optical density, measured at 540 nm, of the suspension of treated cells. The experiments are carried out in triplicates. Dose–response curves are plotted for the different suspensions. These experiments were carried out in triplicate.

#### In vitro heating properties of magnetosome chains, M-uncoated, M-Chi, M-Neri, and M-PEI suspensions brought into contact with GL-261 and RG-2 cells and exposed (or not) to an AMF

GL-261 and RG-2 cells are seeded on a Petri dish of 35 mm in diameter at a density of 2.5 × 10^5^ cells per dish and incubated for 24 h at 37 °C. The culture medium is removed and replaced by 1 mL of culture medium containing, or not, a suspension with 1 mg in iron of magnetosome chains, M-uncoated, M-Chi, M-Neri, or M-PEI. The different assemblages of cells and nanoparticles are then exposed, or not for the control, to an AMF of frequency 198 kHz and average field strength of 34 mT applied during 30 min. The variation of temperature with time of the different mixtures is measured using an infra-red camera (*EasIRTM*-*2, Optophase*) positioned 30 cm above the coil generating the AMF. The infra-red camera measures the spatial temperature distribution in the Petri dish and enables an estimate of the minimum, maximum and average temperature. Increase of the average temperature after 30 min of AMF application and average specific absorption rate deduced from the heating curves are designated by ΔT and SAR, respectively. The SAR is calculated using the relation: SAR = C_water_(ΔT/δt)/X_Fe_, where C_water_ = 4.2 J/(g K) is the specific heat capacity of water, X_Fe_ is the mass of iron in magnetosome (g) per mL, and ∆T/δt is the initial slope of the temperature variation with time. These experiments were carried out in triplicate.

#### Cytotoxicity of magnetosome chains, M-uncoated, M-Chi, M-Neri, and M-PEI suspensions brought into contact with GL-261 and RG-2 cells and heated (or not) at 43–46 °C for 30 min, by applying an AMF of 198 kHz and strength adjusted to 34–47 mT

GL-261 and RG-2 cells are seeded on a Petri dish of 35 mm in diameter at a density of 2.5 10^5^ cells per dish and incubated for 24 h at 37 °C. The culture medium is removed and replaced by 1 mL of complete medium containing, or not for the control, 1 mg of iron in MC, M-uncoated, M-Chi, M-Neri, and M-PEI. The different assemblages of cells and nanoparticles are then exposed, or not for the control, to an AMF of 198 kHz and strength adjusted to 34–47 mT depending on the nanoparticles to reach temperatures between 43 and 46 °C for 30 min. Following application of the AMF, cells are washed with a PBS solution and incubated during 24 h with 2 mL of culture medium at 37 °C and 5% CO_2_. In order to harvest the cells, 500 μL of Trypsin–EDTA are added to the adherent cells. Then 2 mL of liquid medium is added to the harvested cells to homogenize the suspension. In order to evaluate the percentage of living cells, 5 μL of propidium iodide (PI, 20 µg/mL) are added to 500 µL of the cells in suspension in each sample. Since PI only penetrates in dead cells, measurement of its fluorescence provides an estimate of the percentage of dead cells. From this estimate, the percentage of living cells is deduced. In order to measure the fluorescence of PI, cells are analyzed in a flow cytometer (*Beckton Dickinson FACS Calibur 3C*), which contains an argon laser with an emission at 488 nm that excites PI and a FL3-H detector able to detect PI fluorescence. 1 × 10^5^ cells per sample are measured to determine the percentage of living cells. These experiments were carried out in triplicate.

#### Quantity of iron coming from the different magnetosomes internalized in GL-261 and RG-2 cells

5.10^4^ GL-261 and RG-2 cells in 300 µL of culture medium are seeded on µ-Slide 8 Well (80827 from Ibidi^®^) grown during 24 h at 37 °C in 5% CO_2_ atmosphere. After incubation, 200 µL of culture medium contained in each well are removed and 30 µL of magnetosome chains, M-uncoated, M-Chi, M-Neri, and M-PEI, at 10 mg/mL, are introduced in each well. Each well is exposed, or not for the control, to an AMF of 198 kHz and strength adjusted manually to 34–47 mT, depending on the nanoparticle, to reach a temperature of 43–46 °C during 30 min. Following the treatment, cells are washed two times with PBS and it is then verified using an optical microscope that large quantity of magnetosomes or magnetosome aggregates are not at the cell surface. This experiment therefore determines the quantity of magnetosomes internalized in cells, iron being present either as crystallized or dissolved iron. In order to harvest the cells, 100 μL of Trypsin–EDTA are added to GL-261 or RG-2 adherent cells. Then 200 µL of liquid medium are added to the harvested cells to homogenize the suspension. Cells are counted and then used to quantify the quantity of iron per cell. Cells brought into contact with magnetosome chains, M-uncoated, M-Chi, M-Neri, and M-PEI are first harvested by centrifugation at 4000 rpm during 20 min. The supernate is then removed and the cells are re-suspended in 250 µL of HCl: HNO_3_ (3:1, v:v) to dissolve the iron oxide into Fe^2+^ and Fe^3+^ ions. The solution of HCl: HNO_3_ (3:1, v:v) is mixed with hydrogen peroxide to produce all Fe^3+^ ions then complexed with potassium thiocyanate, whose quantity is determined by absorbance at 476 nm using a *UviLine 9400 Secomam* spectrophotometer. Solutions containing various concentrations of FeCl_3_ mixed with potassium thiocyanate (2 M) are prepared for calibration. These experiments were carried out in triplicate.

## Results

In this study, coated magnetosome minerals are synthesized according to the following four steps: (i) MSR-1 magnetotactic bacteria are first cultivated in a fermenter, (ii) Chains of magnetosomes are isolated from magnetotactic bacteria, (iii) chains of magnetosomes with biological membrane are purified to yield uncoated magnetosome minerals and, (iv) these minerals are covered with one of three coating substances, i.e. chitosan, neridronate, or polyethyleneimine.

### Categorization of the different magnetosome suspensions as medical devices of class III

Magnetosomes presented here are intended for use in the magnetic hyperthermia treatment of tumours. Their mode of action therefore comes from heat and does not involve a dominant pharmacological, metabolic, or immunologic effect. Magnetosomes are therefore categorized as implantable medical device of class III in agreement with the classification of other nanoparticles such as Nanotherm [25], or NBTXR3 [26], used for the treatment of tumors under radiation. In this study, ISO 10993 medical device standards are thus followed to assess magnetosome biocompatibility.

### Properties of magnetosome chains (MC)

**Fig. 1:**
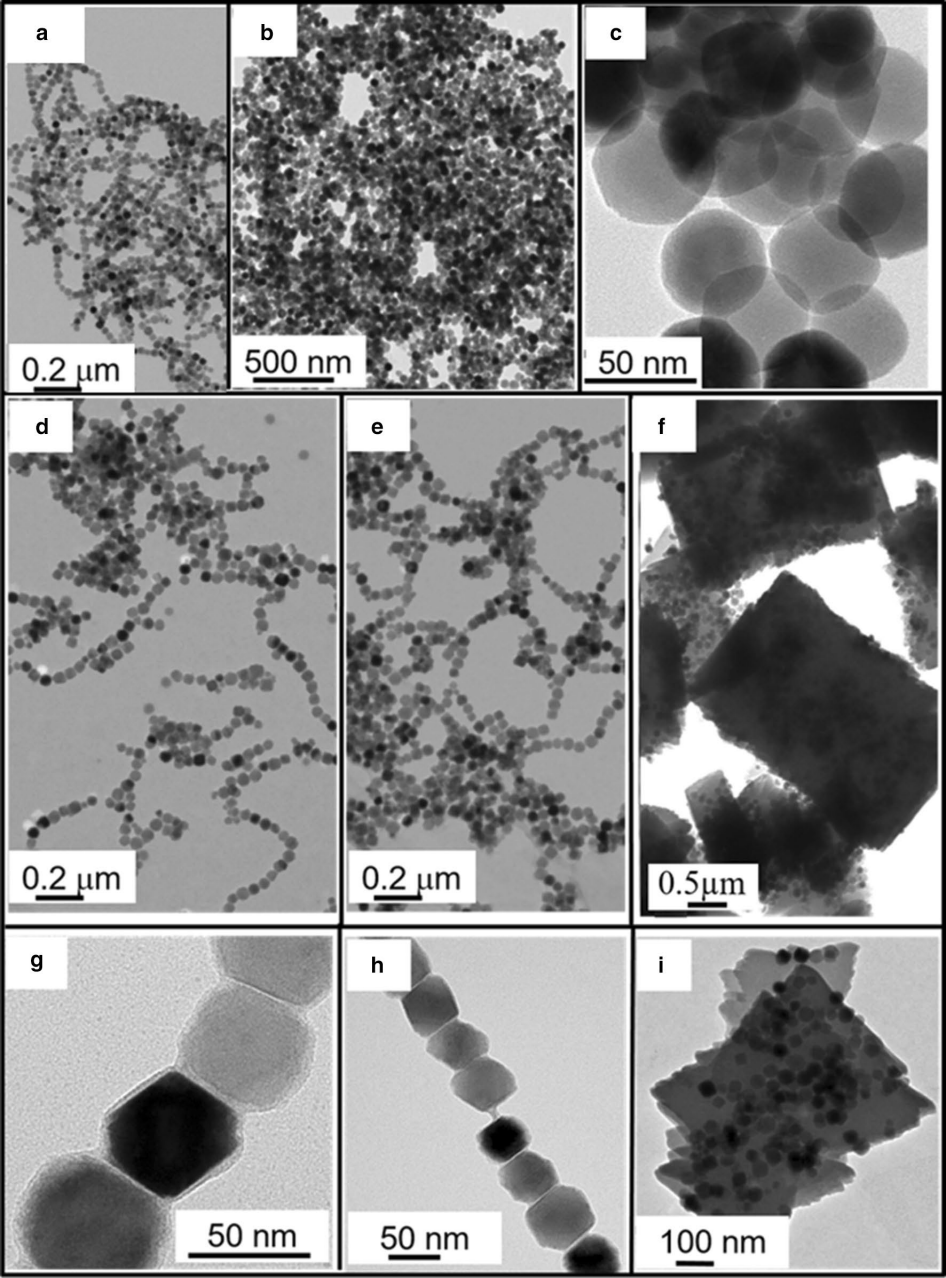
Transmission electron microscopy images of **a** chains of magnetosome post lysis, MC, **b**, **c** uncoated magnetosome minerals, M-uncoated, **d**, **g**, magnetosomes minerals coated with Polyethyleneimine, M-PEI, **e**, **h**, Chitosan, M-Chi, **f**, **i**, or Neridronate, M-Neri

**Fig. 2:**
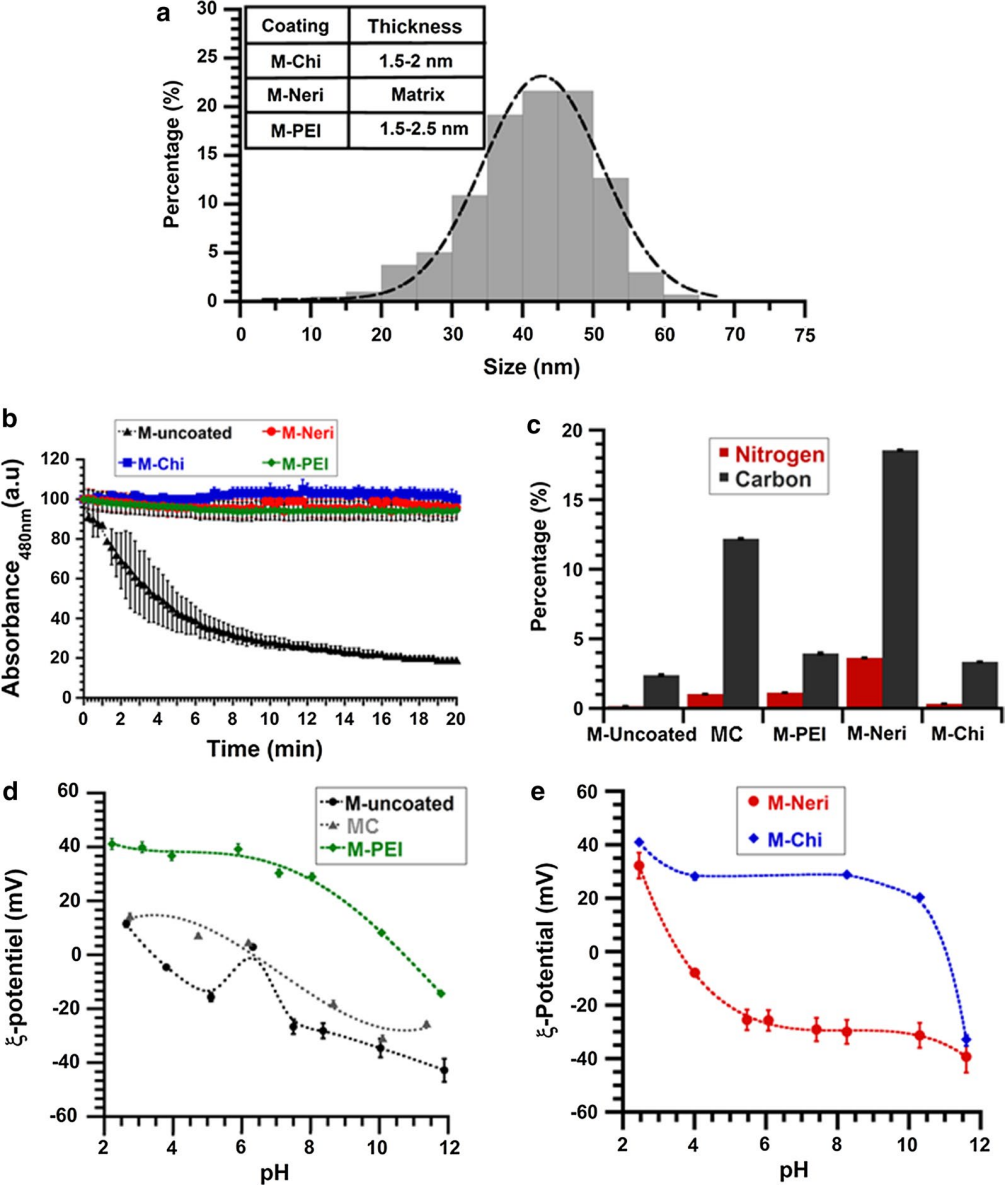
**a** Size distribution of uncoated magnetosome minerals and coating thicknesses of M-Chi, M-Neri and M-PEI indicated in the *inset*, **b** variation of the absorbance, measured at 480 nm, of a suspension containing 1 mg/mL in iron of uncoated and coated magnetosome minerals as a function of time, where the absorbance is normalized by the absorbance at the beginning of the measurements, **c** percentage in weight of carbon and nitrogen, measured with a CHNS, of magnetosome chains, uncoated and coated magnetosome minerals, **d** zeta potential measurements of suspensions of MC, M-uncoated, and M-PEI, as a function of pH, **e** zeta potential measurements of suspensions of M-Neri and M-Chi as a function of pH

**Fig. 3:**
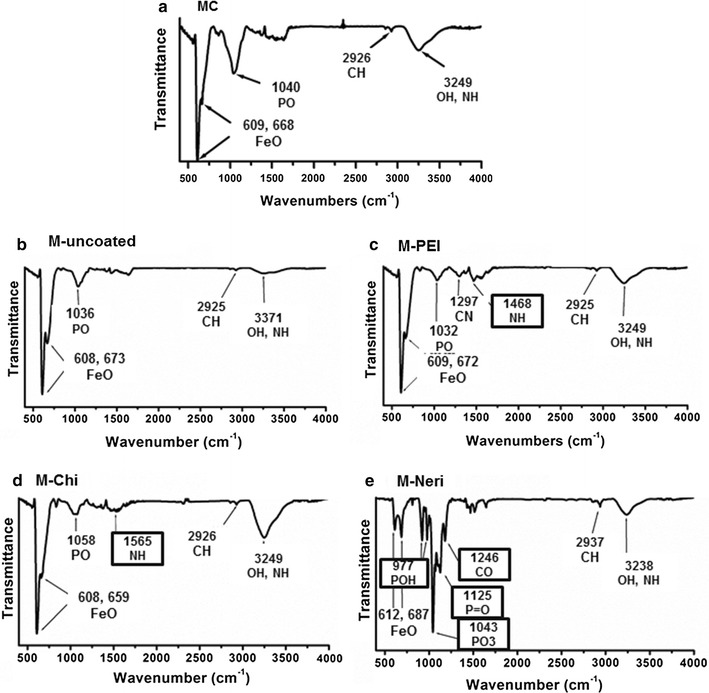
Fourier transform infrared spectra of powders containing lyophilized powders of MC (**a**), M-uncoated (**b**), M-PEI (**c**), M-Chi (**d**) and M-Neri (**e**). Peaks whose position is surrounded are those arising from the coating substances

Chains of magnetosomes extracted from the magnetotactic bacterial cells and not further purified are designated as MC. Figure 1a shows a TEM image of MC extracted from magnetotactic bacteria by heating in a sonicating bath a suspension of magnetotactic bacteria at 80 °C during 2 h in the presence of 5 M NaOH without any further treatment. Despite of their appealing features, such as strong magnetic properties and arrangement in chains [27], such magnetosomes can hardly be used for medical applications due to their high endotoxin concentration of 2000–12,000 EU per mL per mg of iron comprised in nanoparticles, that makes them pyrogenic (Table 1). In addition, the percentage of organic carbon surrounding those magnetosomes deduced from CHNS measurements is significant at 12 ± 0.04% (Fig. 2c), confirming the presence of an organic layer surrounding the magnetosome mineral core in MC [28]. Magnetosomes are analyzed by FT-IR measurements, that reveal a series of FT-IR peaks, shown in Fig. 3a, a Fe–O stretching vibration band at 609–668 cm^−1^ attributed to iron oxide, a peak at 900–1200 cm^−1^ corresponding to the P–O bending vibration bands and two peaks at 2850–2950 and 3200–3600 cm^−1^ representing N–H bending vibration bands of proteins bound to the magnetosomes [29], and C–H stretching vibration bands of other organic material at magnetosome surface [30], respectively. The surface charge of MC decreases with increasing pH to reach a negative value of −10 ± 0.3 mV at pH 7 (Fig. 2d).

### Properties of uncoated magnetosome minerals

In order to remove endotoxins from the magnetosomes, MC are further purified with heat treatment in the presence of different detergents (1% Triton X-100, 1% SDS and phenol) and heated at 60 °C under sonication. Purification removes most of the organic material surrounding the magnetosome mineral core and yields uncoated magnetosomes. The composition of uncoated magnetosomes is determined to be maghemite, since in the saturating isothermal remanent magnetization spectrum of such mineral presented elsewhere, the Verwey transition is not observed [31, 32]. Purification enables to decrease the endotoxin concentration by a factor of 100 compared to MC down to 18–130 EU per mL per mg of iron (Table 1), as estimated with the LAL test. In M-uncoated, the percentages of carbon and Nitrogen deduced from CHNS measurements are 2.4 ± 0.06 and 0.2 ± 0.002% respectively, lower than those of 12 ± 0.04 and 1 ± 0.01% measured for the MC unpurified magnetosome chains, in which the mineral cores are surrounded by an organic layer [33]. The low quantity of organic material remaining after purification is further highlighted by the FT-IR spectrum of M-Uncoated, which is presented in Fig. 3b, and displays the peak of the Fe–O stretching vibration band at 608-675 cm^−1^ attributed to iron oxide and three weaker peaks also observed in the spectrum at 1200–900, 2850–2950 and 3200–3600 cm^−1^ (Fig. 3a), corresponding to the P–O bending vibration band, C–H stretching vibration band and –NH_2_ bending vibration band, respectively.

Despite the fact that M-uncoated appear relatively pure, Fig. 1b and c, which show TEM images of M-uncoated suspension deposited and dried on top of a carbon grid, reveal that these magnetosomes are aggregated, which may be due to their large sizes ranging from 10 to 65 nm, leading to strong mutual magnetic attraction. As shown in Fig. 2b, the absorbance of a suspension of M-uncoated decreases by 50 ± 1% in 4 min, indicating the low stability and fast sedimentation of M-uncoated in aqueous suspension. Furthermore, the formation of such aggregates is suggested by the unusual zeta potential variation around pH 7, i.e. an increase followed by a decrease between pH 5 and pH 7.5 (Fig. 2d) [30, 33], leading to two isoelectric points (PI), at pH 4.5 and 6.5.

### Properties of coated magnetosome minerals M-Chi, M-PEI, and M-Neri

Administration of a medical product requires a good stability in suspension. For medical applications, aggregation should also be avoided to prevent complications due for example to thrombosis of blood vessels [34]. In this study, magnetosome minerals are coated with three different substances to lead a highly stable suspension: polyethyleneimine [35], chitosan [36], or neridronate [37]. These substances have also been chosen to favor interactions with the mineral core using chelating properties of phosphonic acids in neridronate or the presence of polymer in chitosan and PEI that promotes adsorption at magnetosome surface.

The presence of the different coating substances at the surface of magnetosome minerals is first revealed by transmission electron microscopy (TEM) measurements, which show a 1.5–2 nm thick homogeneous coating surrounding the magnetosome mineral core in M-PEI (Fig. 1g) and M-Chi (Fig. 1h). In M-PEI and M-Chi most of magnetosome also appear organized in chains containing ~7 magnetosomes on average. For M-Neri, Fig. 1(i) shows that a quasi-parallelepipedic matrix of average surface 3.5 µm^2^ in 2D projections surrounds each magnetosome mineral core. Second, FT-IR spectra show a series of peaks, which are not present in the spectrum of M-uncoated, and are therefore a signature of the coating (Fig. 3c–e). Such peaks are observed at 1468 cm^−1^ (Fig. 3c), corresponding to the N–H bending vibration band of PEI [38], at 1565 cm^−1^, attributed to the N–H bending vibration band of chitosan (Fig. 3d) [39], and at 977, 1043 and 1125 cm^−1^ (Fig. 3e), which can be assigned to the P–OH and P=O bands of neridronate [40]. Third, Fig. 2c shows that the percentage of carbon, measured with the CHNS, is higher than in M-uncoated by ~1.6 ± 0.06, ~1 ± 0.02 and ~16 ± 0.04% in M-PEI, M-Chi, and M-Neri, respectively. From CHNS analysis, we deduced that the percentages of carbon were 54.1 ± 0.1, 31.3 ± 0.4 and 26.1 ± 0.3% for PEI, Chi and Neri respectively. Furthermore, the ratio between the percentages of carbon at magnetosome surface and in the coating agent represent the percentage of coating agent at magnetosome surface, considering that 100% of carbon at the surface of coated comes from the coating agent. The percentages of coating agent at magnetosome surface were estimated as 7.09% for M-PEI, 10.66% for M-Chi and 71.13% M-Neri. This result agrees with previous measurements, which suggest that M-PEI and M-Chi are surrounded by a thin layer while M-Neri are covered by a thick matrix. The presence of coating substances is also suggested by ξ-potential measurements as a function of pH varied between pH 2 and pH 11 for aqueous suspensions containing M-PEI, M-Chi and M-Neri (Fig. 2d, e). On the one hand, M-PEI and M-Chi are positively charged at pH < 11 (Fig. 2d, e), suggesting the presence of protonated amino groups at the surface of these coated magnetosome minerals, which should appear for PEI and chitosan coatings at physiological pH. On the other hand, M-Neri are negatively charged at physiological pH, which is expected due to the presence of bisphosphonate groups in Neridronate.

The different coatings surrounding magnetosome minerals in M-PEI, M-Chi and M-Neri lead to more stable suspensions in water than in M-uncoated, as observed in Fig. 2b that shows a decrease in the absorption of these suspensions of less than 4 ± 0.16% after 20 min, and as revealed by zeta potential measurements presented in Fig. 2d and e that indicate isoelectric points (PI) of 10.5, 11 and 3.5, for M-PEI, M-Chi, and M-Neri, respectively, three values that differ significantly enough from 7 to illustrate strong electrostatic repulsive forces between nanoparticles leading to colloidal stability in water [41]. Furthermore, in cell culture medium (RPMI and DMEM), Additional file 1: Figure S4a and b also show that M-PEI, M-Chi and M-Neri lead to more stable suspensions than M-uncoated but the decrease with time of the absorbance of all magnetosome suspensions is much more rapid than in water. This may be due to the presence of proteins and electrolytes in cell culture media that favors nanoparticle sedimentation [42].

### Cytotoxicity of M-Chi, M-PEI, and M-Neri in the absence of alternate magnetic field application on 3T3 healthy cells compared with that of MC and uncoated magnetosomes

**Fig. 4:**
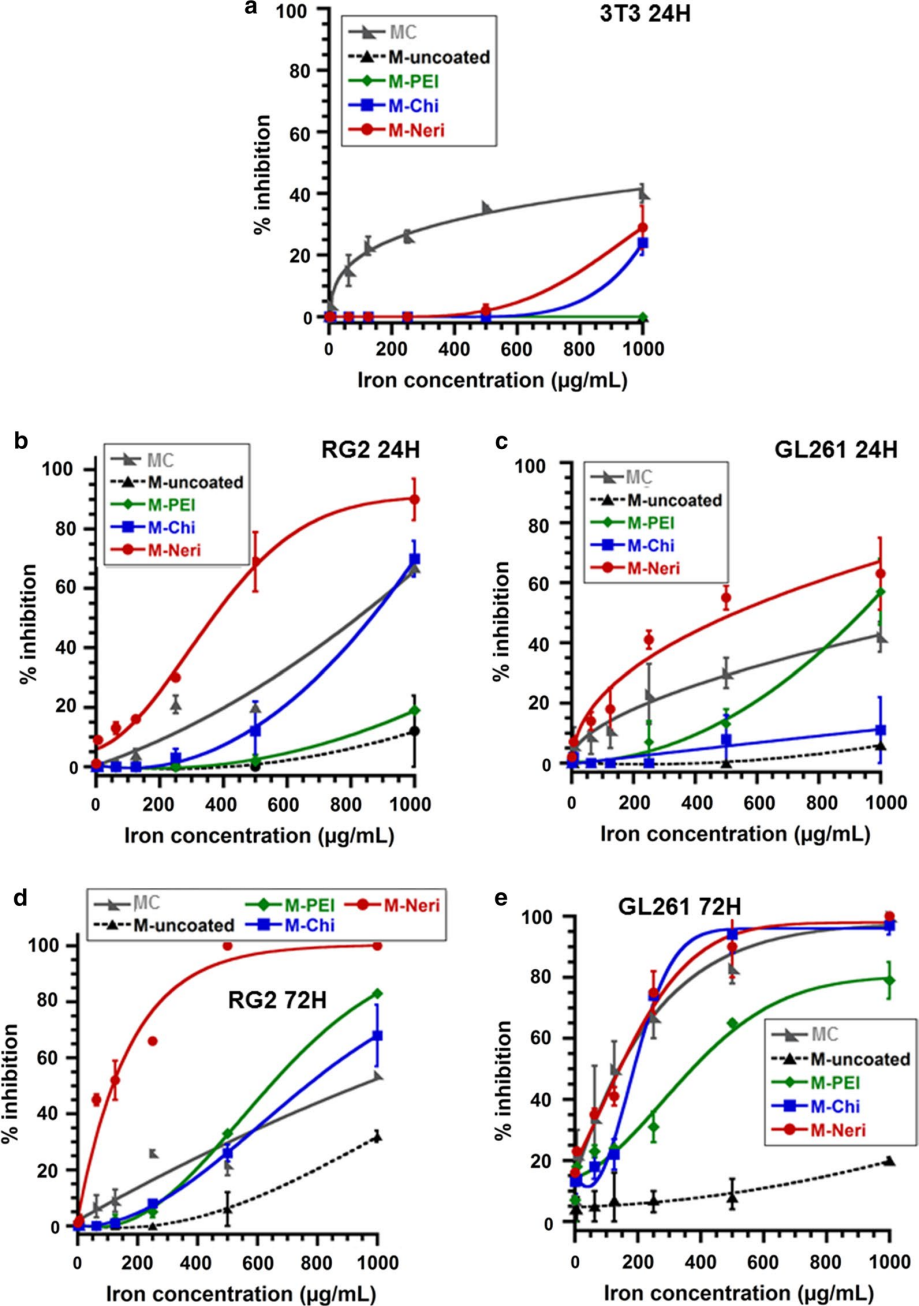
Percentage of cell inhibition as a function of the various magnetosome concentration, measured in µg of iron per mL, of 3T3 cells incubated with the various magnetosomes during 24 h (**a**), of RG-2 cells incubated with the various magnetosomes during 24 h (**b**), of GL-261 cells incubated with the various magnetosomes during 24 h (**c**), of RG-2 cells incubated with the various magnetosomes during 72 h (**d**), and of GL-261 cells incubated with the various magnetosomes during 72 h (**e**)

To further examine the biocompatibility of M-Chi, M-PEI, and M-Neri, and compare it with that of magnetosome chains and uncoated magnetosomes, the different types of magnetosomes were incubated with the mouse fibroblast healthy 3T3 cells during 24 h. Cytotoxicity against 3T3 cells induced by the presence of these magnetosomes was measured using a neutral red (NR) assay according to the ISO 10993-5 standard. Figure 4a shows the percentage of cell inhibition as a function of magnetosome concentration, varied between 16 and 1 mg/mL in iron. While for MC, the percentage of cell inhibition rapidly increases with increasing magnetosome concentration to reach 40 ± 3% at 1 mg/mL, it remains close to 0% for magnetosome concentration below 500 µg/mL and lower than 30% over the whole range of tested concentrations for M-uncoated, M-PEI, M-Chi and M-Neri. This suggests that purification for M-uncoated or purification followed by re-coating for M-PEI, M-Chi, and M-Neri, has significantly reduced magnetosome cytotoxicity to yield coated magnetosome minerals with an average percentage of cell inhibition, which is lower than 30%, the threshold below which medical devices are usually considered non-cytotoxic according to the ISO 10993-5 standard.

### Cytotoxicity of coated magnetosome minerals in the absence of alternate magnetic field application on RG-2 and GL-261 cells compared with that of MC, uncoated magnetosomes, and free coating agents

Cytotoxicity of magnetosome chains, uncoated and coated magnetosome minerals, was also estimated on RG-2 and GL-261 glioblastoma cells using the MTT assay, which provides the same information as the neutral red assay. Percentages of cell inhibition after 24 h of incubation in the presence of the different nanoparticles, at concentration varied between 15.6 and 1000 µg/mL, are presented in Fig. 4b and c for RG-2 and GL-261 cells, respectively. Depending on nanoparticle type, magnetosomes appear to be either equivalently or more cytotoxic towards RG-2 and GL-261 tumor cells than towards 3T3 healthy cells (Fig. 4a–c). At 24 h, the largest cytotoxicity difference between tumor and healthy cells is observed with M-Neri that reach 90 ± 7 and 63 ± 12% of cell inhibition on RG-2 and GL-261 cells, respectively, (Fig. 4b, c) compared with only 29 ± 7% on 3T3 cells (Fig. 4a). When the same nanoparticles are incubated during 72 h in the presence of RG-2 and GL-261 cells, the percentage of cell inhibition is significantly larger than for 24 h of incubation (Fig. 4b–e). The less cytotoxic magnetosomes appear to be uncoated magnetosomes and M-PEI, which do not reach 100% of cell inhibition in any of the tested conditions. MC and M-Chi are relatively non-cytotoxic, only reaching 100% of cell inhibition when they are incubated with GL-261 cells during 72 h at concentrations above 1 mg/mL. M-Neri seem to produce the largest cytotoxic, reaching 100% of cell inhibition when they are incubated during 24 h with RG-2 cells or during 72 h with RG-2 and GL-261 cells (Fig. 4b, d, e). As a whole, M-PEI, M-Chi, and M-Neri, appear to produce higher cytotoxicity towards RG-2 and GL-261 cells than towards 3T3 cells and this enhancement is globally more pronounced than for M-uncoated and MC (Fig. 4a–e).

We have then compared the cytotoxicity of coated magnetosomes with that of free coating agents. We have estimated that for 1 mg of magnetosomes in iron, the quantities of coating agents at magnetosome surface are 71, 107, and 711 µg for M-PEI, M-Chi, and M-Neri, respectively. When GL-261, RG-2, and 3T3 cells are incubated during 24 h with these quantities of coating agents, the percentage of inhibition is lower than 3% for the 3 cell lines (Additional file 1: Figure S1a). It indicates that the cytotoxicity of the coating agent is enhanced when it is associated to magnetosomes, possibly due to more interactions of the coating agents with cells when they are associated to the magnetosomes.

### Specific absorption rates, cellular internalization and percentage of cellular death of uncoated magnetosomes, MC, M-Neri, M-PEI, and M-Chi

**Fig. 5:**
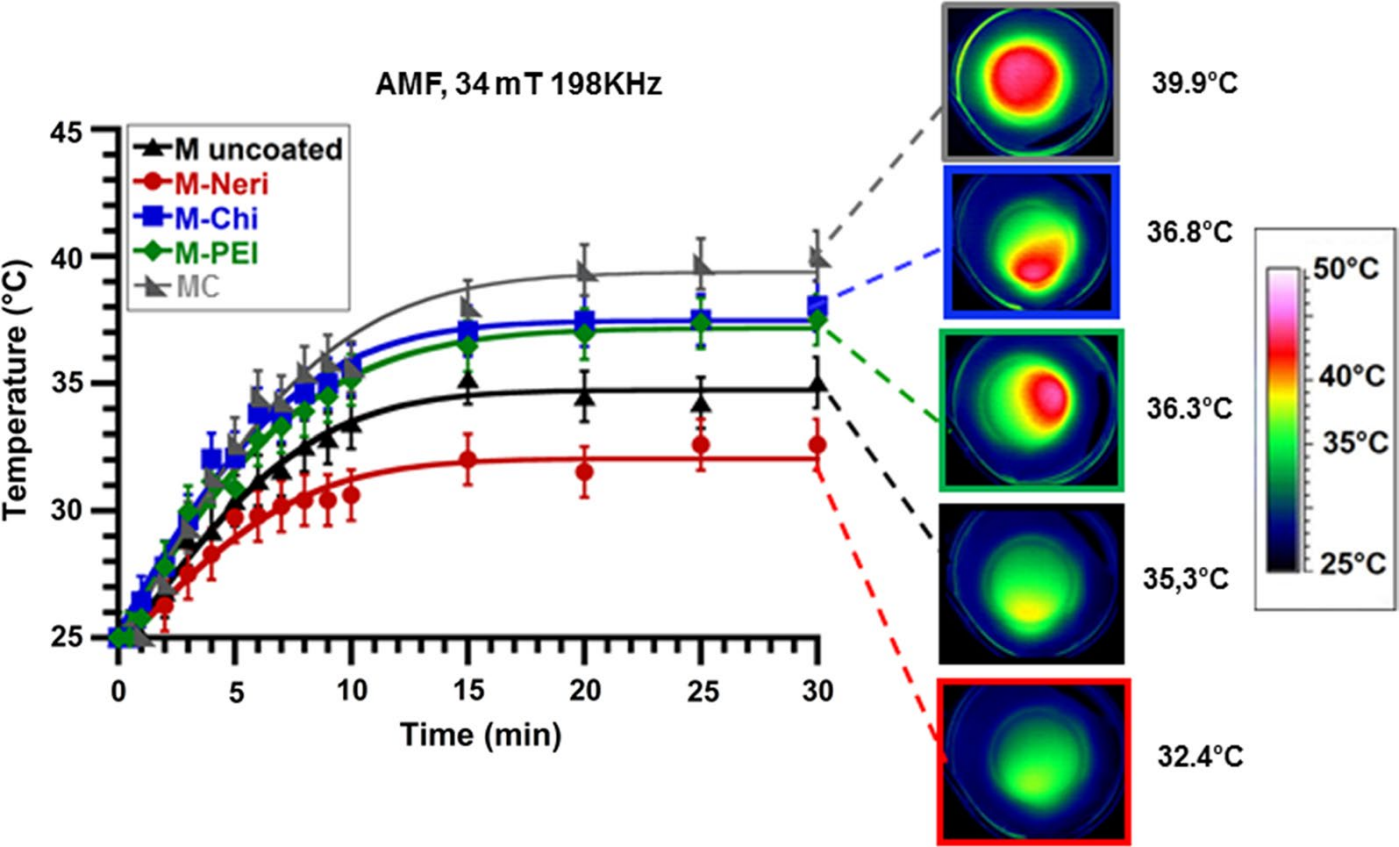
Variation with time of the average temperature of GL-261 cells brought into contact with 1 mg/mL of MC, M-uncoated, M-Neri, M-Chi or M-PEI, exposed to an AMF of frequency 198 kHz and average strength 34 mT applied during 30 min. Each point in the plot represents the average spatial temperature of the cells with magnetosomes in the Petri dish measured with an infra-red camera. *On the right-hand side*, spatial temperature distribution, measured with an infra-red camera, of GL-261 cells brought into contact with 1 mg/mL of M-uncoated, M-Neri, M-Chi, or M-PEI, after 30 min of application of an AMF of frequency 198 kHz and average strength 34 mT

1 mg/mL in iron of suspensions containing the different types of magnetosomes, which is the lowest concentration that can induce a detectable temperature increase, were brought into contact with GL-261 and RG-2 cells and exposed to a magnetic treatment during which an AMF of frequency 198 kHz and average field strength 34 mT is applied during 30 min. The average temperature is measured over the whole Petri dish containing the cells and the various nanoparticles. Figure 5 shows that it increases from 25 ± 2 °C before magnetic treatment to maximum temperatures, T_max_, of 40 ± 2, 36 ± 2, and 37 ± 2 °C for MC, M-PEI, and M-Chi, respectively, higher than T_max_ of 35 ± 2 and 32 ± 2 °C found for uncoated magnetosomes and M-Neri, respectively. Using the relation SAR = C_water_(ΔT/δt)/X_Fe_, where C_water_ = 4.2 J/(g K) is the specific heat capacity of water, X_Fe_ = 0.001 g/mL is the quantity of magnetosomes in iron (g) per mL and ΔT/δt are the initial slopes of the average temperature variation with time, 0.017 ± 0.0006 °C/s < ΔT/δt < 0.03 ± 0.0012 °C/s, deduced from the plots of Fig. 5 (Table 2), average SAR are estimated as 86 ± 3.4, 128 ± 5.1, 120 ± 4.7_e_, 125 ± 5 and 72 ± 2.8 W/g_Fe_ for uncoated magnetosomes, MC, M-PEI, M-Chi, and M-Neri, respectively. The highest values of the average SAR and of T_max_ are deduced for MC, M-PEI and M-Chi, indicating that these nanoparticles yield the best heating properties. Furthermore, in vitro anti-tumor efficacy of the various magnetosomes under AMF application is estimated by measuring the decrease in the percentage of GL-261 and RG-2 living cells after the following two conditions have been applied: (i) GL-261 and RG-2 cells are brought into contact during 30 min with 1 mg/mL in iron of the different types of magnetosomes at 37 °C in the absence of AMF for the control and (ii) GL-261 and RG-2 cells are treated as in (i) and then immediately exposed during 30 min to an AMF of frequency 198 kHz and average field strength of 47 mT (uncoated magnetosomes), 34 mT (MC), 35 mT (M-PEI), 43 mT (M-Chi), and 47 mT (M-Neri), to maintain the temperature of the assemblies at 43 °C to 46 °C during treatment.

**Table 2:** Initial slopes of the plots shown in Fig. 5, ΔT/δt, measured in °C/s, specific absorption rates (SAR), measured in Watt per gram of iron, deduced from the values of ΔT/δt, maximum temperature (T_max_) over the whole petri dishes observed after 30 min of heating deduced from Fig. 5

	T_max_ (°C)	ΔT/δt (°C/s)	SAR (W/g_fer_)
MC	40 ± 2	0.030 ± 0.0012	128 ± 3.4
M-uncoated	35 ± 2	0.020 ± 0.0008	86 ± 5.1
M-Chi	37 ± 2	0.029 ± 0.001	125 ± 5.0
M-PEI	36 ± 2	0.028 ± 0.0011	120 ± 4.7
M-Neri	32 ± 2	0.017 ± 0.0006	72 ± 2.8

**Fig. 6:**
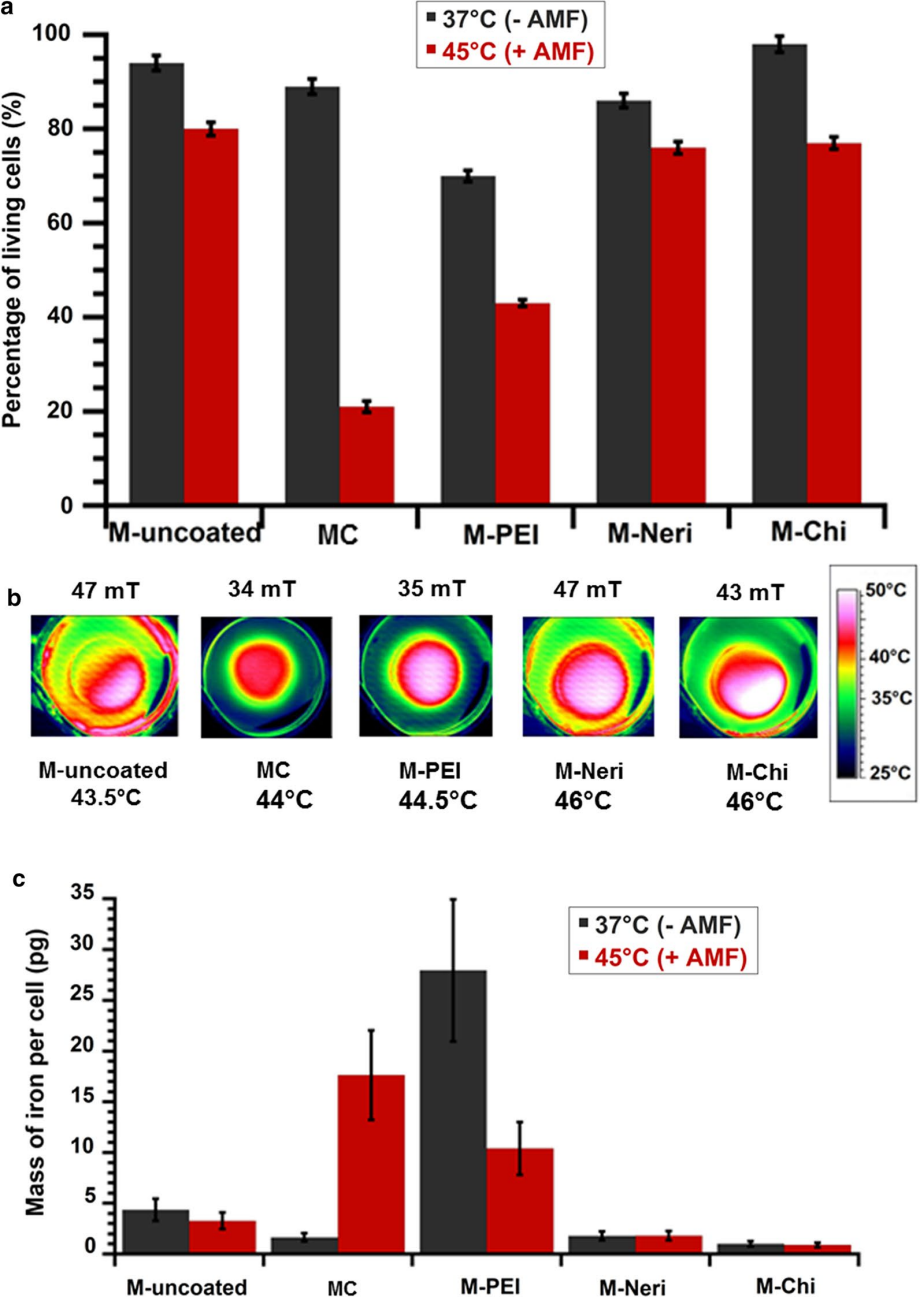
**a** Percentage of living GL-261 cells (%) when these cells are brought into contact with 1 mg/mL of MC, M-uncoated, M-Neri, M-Chi, and M-PEI, and either maintained at 37 °C during 30 min without AMF treatment,* black columns*: 37 °C (−AMF), or exposed during 30 min to an AMF of frequency 198 kHz and strength varied between 34 and 47 mT to maintain temperature at between 43 and 46 °C during 30 min,* red columns*: 45 °C (+AMF). **b** Spatial temperature distribution, measured with an infra-red camera, of GL-261 cells brought into contact with 1 mg/mL of MC, M-uncoated, M-Neri, M-Chi or M-PEI and exposed during 30 min to the same AMF as in **a**. **c** Quantity of iron coming from magnetosomes, which is internalized in each GL-261 cell when GL-261 cells are brought into contact with 1 mg/mL of MC, M-uncoated, M-Neri, M-Chi, or M-PEI, and exposed during 30 min to the same AMF as in **a**

Comparing conditions (i) and (ii), for GL-261 and RG-2 cell lines the percentage of living cells decreases from 94 ± 1.6% down to 80 ± 1.4% (GL-261) and from 67 ± 1.3% down to 54 ± 1.1% (RG-2) for uncoated magnetosomes, from 86 ± 1.6% to 20 ± 1.2 (GL-261) and from 77 ± 1.7 to 64 ± 1.6% (RG-2) % for MC, from 70 ± 1.2 to 43 ± 0.7% (GL-261) and from 58 ± 1.0 to 47 ± 0.7% for M-PEI, from 86 ± 1.7 to 76 ± 1.3% (GL-261) and from 72 ± 1.2 to 62 ± 1.0% (RG-2) for M-Neri, and from 98 ± 1.5 to 77 ± 1.3% (GL-261) and from 86 ± 1.5% to 69 ± 1.1% (RG-2) for M-Chi (Fig. 6a; Additional file 1: Figure S2a). It indicates that the various magnetosomes all lead to enhanced GL-261 and RG-2 cell death after one application of the AMF.

With regard to the quantity of internalized magnetosomes in GL-261 cell line, in the absence of AMF application, it is either relatively significant with GL-261 cell line for M-PEI and MC at 28 ± 7 and 6 ± 1.5 pg of iron per cell, respectively, or low for uncoated magnetosomes, M-Neri and M-Chi at 1 ± 0.2 to 4.4 ± 1.1 pg of iron per cell. Comparing conditions (i) and (ii), the quantities of internalized magnetosomes remain either unchanged for M-uncoated, M-Neri and M-Chi at 1 ± 0.2 to 3.3 ± 0.8 pg of iron per cell, increases for MC from 6 ± 1.5 pg up to 18 ± 4.5 pg of iron per cell, or decreases for M-PEI from 28 ± 7 pg down to 10 ± 3 pg of iron per cell. As observed with the GL-261 cell line, when the AMF is applied the quantity of nanoparticles internalized in RG-2 cells remains relatively unchanged for M-Uncoated at 0.7 ± 0.14 pg or decreases by 0.71 ± 0.14 to 0.35 ± 0.07 pg. for M-PEI (Additional file 1: Figure S3b). M-Neri and M-Chi, seem to internalize more in RG-2 than GL-261 cells, which suggests that internalization rate may not only depend on AMF application but also on cell type.

## Discussion

Although significant work has been carried out in the field of magnetic hyperthermia, which can certainly lead to improved efficacy compared with existing cancer treatments, it seems that nanoparticles previously used were either suboptimal with regard to heating efficacy or possessed high SAR values but lacked biocompatibility, due for example to a composition that included toxic transition metals such as cobalt [42].

In this study, we used an original nanoparticle synthesis method, which involves a specific strain of bacteria called magnetotactic bacteria instead of a chemical synthesis and we were able to fabricate nanoparticles, i.e. coated magnetosome minerals (M-PEI, M-Neri, and M-Chi), which are characterized by enhanced heating and biocompatibility properties. Indeed, these nanoparticles possess a low endotoxin concentration of 12–203 EU per mL per mg of iron comprised in the nanoparticles. To verify that these endotoxin concentrations deduced from the LAL assay corresponded to non-pyrogenic suspensions, we carried out a rabbit test by administering in the ear of rabbits a suspension of coated magnetosomes minerals with an endotoxin concentration, of 80 EU/mg/mL. It did not induce any fever among rabbits indicating that these endotoxin concentrations corresponded to non-pyrogenic suspensions [32].

Furthermore, at a concentration of 22 µg of magnetosomes in iron per mL, which corresponds to the concentration recommended for testing the biocompatibility od medical devices (6 cm^2^/mL) [43], they yield a percentage of 3T3 cell inhibition, which is below 30%, indicating that they are biocompatible according to the ISO 10993-5 standard. They may also guarantee a safe treatment since they appear to be more cytotoxic towards GL-261 or RG-2 glioma cells than towards 3T3 healthy cells [44].

Intratumor administration may remain the best administration route for glioblastoma treatment since it enables to concentrate nanoparticles in the tumor and avoid side effects that may arise from a systemic injection. For this reason, we used two tumor cell lines (RG-2 and GL-261) with which nanoparticles are expected to mainly interact to assess cytotoxicity, internalization, and heating properties of the nanoparticles in the presence of the AMF.

Efficacy of M-PEI, M-Neri, and M-Chi for the magnetic hyperthermia treatment of tumors was further examined in vitro. M-PEI and M-Chi produce the largest decrease in GL-261 cell viability under AMF application (21–27% compared to 10% for M-Neri) and lead to the highest SAR value (120–125 W/g_Fe_ compared with 72 W/g_Fe_ for M-Neri). Improved properties of M-Chi and M-PEI compared with those of M-Neri may be due to different types of magnetosome organization between M-Chi and M-PEI on the one hand and M-Neri on the other hand. M-PEI and M-Chi appear organized in chains, similarly to what is observed for magnetosomes naturally synthesized by and extracted from magnetotactic bacteria, a type of organization that is difficult to obtain using a chemical synthesis [45]. This may be due to an average smaller size or a multi-domain structure of chemically synthesized nanoparticles than in M-PEI and M-Chi. The organization in chains of M-PEI and M-Chi may favor cellular destruction either through specific nanoparticle mechanical or thermal interactions with cell membranes or via nanoparticle intracellular heating, notably depending on the level of cellular internalization of these chains [46]. In striking contrast, due to a coating reaction, which is carried out at acidic pH under sonication and heating, bisphosphonate polymerization has likely occurred together with the preparation of M-Neri, resulting in the embedment of magnetosome minerals in a matrix [47]. The presence of a thick matrix in M-Neri may decrease both specific interactions with the cell membrane and intracellular heating, which could explain the lower percentage of cell destruction observed in this case. Compared with the GL-261 cell line, we also observed a decrease in cellular viability when the various nanoparticles were incubated during 24 h with the RG-2 cells and exposed to the same AMF although this decrease of 11–17% was globally slightly less pronounced with RG-2 than with GL-261 cells (Additional file 1: Figure S3a).

The low level of cellular internalization observed with and without AMF application in the cases of M-Chi, M-Neri and uncoated magnetosomes may be due to the presence of a specific coating in M-Chi since chitosan with molecular mass at 600–800 kDa and degree of deacetylation at 90% are known to produce a low level of cellular internalization [51], to the formation of aggregates with uncoated magnetosomes and to the abundant and extended matrix surrounding magnetosomes in M-Neri, which may prevent efficient cellular internalization, possibly by endocytosis [52]. The effect of AMF application on cellular interactions is well observed in the case of MC that had rather low rates of cellular internalization in the absence of AMF, a behavior which is expected for negatively charged nanoparticles [53], and by contrast to enhanced cellular internalization in the presence of AMF [54]. For MC, internalization may be influenced both by the presence of a negative charge at nanoparticle surface and by the organization in chains of the nanoparticles. Such properties may indeed increase the coupling between nanoparticles and the AMF and possibly favor endocytosis, a type of behavior that was previously reported for nanoparticles exposed to a non-oscillating magnetic field [55]. Whereas the efficacy of the coupling between the nanoparticles and the magnetic field was previously attributed to nanoparticle high magnetization [55], we believe that nanoparticle surface charge is another important parameter that should be considered to account for such behavior. Most interestingly, M-PEI, which are highly internalized even without AMF, possibly due to the presence of a positively charged transfecting agent (PEI) at magnetosome surface [52], seem to be expelled from cells, following AMF application, possibly through exocytosis [56].

Furthermore, high nanoparticle internalization of up to 20–25 pg per cell were observed in some conditions (MC with AMF or M-PEI without AMF), which could be explained by the high magnetosome concentration of 1 mg/mL incubated with GL-261 cells during 30 min. Indeed, several studies previously reported a high internalization of iron oxide nanoparticles for rather similar nanoparticle concentration and incubation times, e.g. 50–200 pg/cell were internalized in HeLa or fibroblast cells after 1–6 h(s) incubation of 120–600 µg/mL of iron oxide nanoparticles with cells [48, 49]. This suggests an enhanced amount of internalized nanoparticles at high magnetosome concentrations as previously demonstrated with other nanoparticle types [50].

## Conclusions

Magnetosomes synthesized by MSR-1 magnetotactic bacteria were purified to remove most organic material surrounding the crystalized mineral core of the magnetosomes and then stabilized. Uncoated magnetosome minerals with a low percentage of residual organic material originating from magnetotactic bacteria were coated with three different substances: chitosan, polyethyleneimine and neridronate. Those coated magnetosomes are either surrounded by a thin layer of 1.5–2.5 nm leading to minerals organized in chains for M-Chi and M-PEI or embedded in an organic matrix for M-Neri. These coated magnetosome minerals appear to be biocompatible due to: (i) their non-pyrogenicity characterized by low endotoxin concentrations of 12–203 EU per mL per mg in iron, (ii) a sufficient stability in suspension to enable in vivo administration, (iii) an absence of cytotoxicity towards healthy 3T3 cells according to the criteria of the ISO 10993-5 standard, (iv) a larger cytotoxicity towards GL-261 and RG-2 tumor than towards 3T3 healthy cells. Furthermore, when M-PEI and M-Chi are brought into contact with GL-261 cells and heated at 43–46 °C during 30 min by application of an AMF characterized by a frequency of 198 kHz and a strength of 34–47 mT, treatment leads to a significant cell inhibition of 20–30%, which is attributed to the relatively large SAR of 120–125 W/gFe and arrangement in chains of M-PEI and M-Chi. By contrast, under the same conditions, M-Neri yield a lower cell inhibition, 10%, which may be attributed to a lower SAR of 72 W/gFe and to the presence of a thick matrix surrounding magnetosome mineral core that may prevent efficient interactions with cells. As a whole, although a significant percentage of cell inhibition was observed at 1 mg/mL, which may be explained by rapid magnetosome sedimentation in culture growth media, we observed at this concentration a decrease in the percentage of living cells under AMF application for all different types of magnetosomes tested. This result indicates that magnetosomes exposed to AMF enhance cellular toxicity even under conditions of rapid nanoparticle sedimentation. Among the different magnetosomes, due to their biocompatibility, heating and organization properties, M-Chi and M-PEI appear to be good candidates for the treatment of tumor by magnetic hyperthermia. This conclusion is further supported by our in vivo experiments performed on mice bearing breast and glioblastoma tumors, which showed that intratumor administration of pyrogenic and non-pyrogenic magnetosomes followed by AMF application yield full tumor disappearance [57–59].

## Additional file

**Additional file 1: Figure S1.** Percentage of cell inhibition of 3T3 cells brought into contact with the various coating agents for 24 h, (a), of RG-2 cells brought into contact with the various coating agents for 24 h, (b), of GL-261 cells brought into contact with the various coating agents for 24 h, (c), of GL-261 cells brought into contact with the various coating agents for 72 h, (d), and of GL-261 cells brought into contact with the various coating agents for 72 h, (e). **Figure S2.** Percentages of GL-261, RG-2, and 3T3 cell inhibition when these cells are incubated with the various magnetosomes (M-PEI, M-Chi, M-Neri) and coating agents (PEI, Chi, Neri) at a concentration of 1 mg/mL during 24 h (a). Percentages of GL-261 and RG-2 cell inhibition when these cells are incubated with the various magnetosomes (M-PEI, M-Chi, M-Neri) and coating agents (PEI, Chi, Neri) at a concentration of 1 mg/mL during 72 h (b). The data presented in this Figure are extracted from Additional file 1: Figure S1. **Figure S3.** (a), Percentage of living RG-2 cells (%) when these cells are brought into contact with 1 mg/mL of MC, M-uncoated, M-Neri, M-Chi, and M-PEI, and either maintained at 37 °C during 30 min without AMF treatment, black columns: 37 °C (−AMF), or exposed during 30 min to an AMF of frequency 198 kHz and strength varied between 34 and 47 mT to maintain temperature at between 43 and 46 °C during 30 min, red columns: 45 °C (+AMF). (b), Spatial temperature distribution, measured with an infra-red camera, of RG-2 cells brought into contact with 1 mg/mL of MC, M-uncoated, M-Neri, M-Chi or M-PEI and exposed during 30 min to the same AMF as in (a). (c), Quantity of iron coming from magnetosomes, which is internalized in each RG-2 cell when RG-2 cells are brought into contact with 1 mg/mL of MC, M-uncoated, M-Neri, M-Chi, or M-PEI, and exposed during 30 min to the same AMF as in (a). **Figure S4.** Variation of the absorbance, measured at 480 nm, of a suspension containing 1 mg/mL in iron of uncoated and coated magnetosome minerals in (a) RPMI 20% FBS, (b) DMEM 10% FBS as a function of time, where the absorbance is normalized by the absorbance at the beginning of the measurements.
