# Supplementary material for: Biocompatible coated magnetosome minerals with various organization and cellular interaction properties induce cytotoxicity towards RG-2 and GL-261 glioma cells in the presence of an alternating magnetic field
Source: J Nanobiotechnology. 2017 Oct 17;15:74. doi: 10.1186/s12951-017-0293-2 (PMC5646109; doi:10.1186/s12951-017-0293-2)

Biocompatible coated magnetosome minerals with various organization and cellular interaction properties induce cytotoxicity towards RG-2 and GL-261 glioma cells in the presence of an alternating magnetic field.

Yasmina Hamdous^1, 2^, Imène Chebbi^1^, Chalani Mandawala^1,3^, Raphael Le Fèvre^1,4^, François Guyot^3^, Olivier Seksek ^2^, Edouard Alphandéry^1,3*^

^1^Nanobacterie SARL, 36 boulevard Flandrin, 75016, Paris.

*^2^* Laboratoire d'Imagerie et Modélisation en Neurobiologie et Cancérologie (IMNC), Campus Universitaire, Bât. 440, 15 rue Georges Clemenceau, 91406 Orsay Cedex, France.

^3^Institut de minéralogie de physique des Matériaux et de Cosmochimie, UMR 7590 CNRS, Université Pierre et Marie Curie, Muséum National d’Histoire Naturelle, Sorbonne Université, 4 Place Jussieu, 75005, Paris, France.

^4^ Institut de Physique du Globe de Paris, Sorbonne Paris Cité, Université Paris Diderot, UMR 7154 CNRS, 1 rue Jussieu, F-75005 Paris, France.

*CORRESPONDING AUTHOR EMAIL ADDRESS:

edouardalphandery@hotmail.com

**SUPPLEMENTARY INFORMATION**

***Pyrogenicity estimate with the rabbit test (ISO 10993-11):***

A rabbit test was also carried out according to ISO10993-11 by using a suspension containing 5 mg/mL of M-PLL, which was first placed in an ultrasonic bath for 2 minutes for homogenization. 1 ml of this suspension was then diluted in 119 ml of 0.9% NaCl. The temperature of the suspension was maintained at 37 °C for 30 minutes, after which the suspension was homogenized and administered intravenously to one rabbit at a dose of 10 ml/kg. The body temperature of the rabbit was measured every 30 minutes during 3 hours.

**Suppl. Fig. 1:** Percentage of cell inhibition of 3T3 cells brought into contact with the various coating agents for 24 hours, (a), of RG-2 cells brought into contact with the various coating agents for 24 hours, (b), of GL-261 cells brought into contact with the various coating agents for 24 hours, (c), of GL-261 cells brought into contact with the various coating agents for 72 hours, (d), and of GL-261 cells brought into contact with the various coating agents for 72 hours, (e).

**Suppl. Fig. 2:** Percentages of GL-261, RG-2, and 3T3 cell inhibition when these cells are incubated with the various magnetosomes (M-PEI, M-Chi, M-Neri) and coating agents (PEI, Chi, Neri) at a concentration of 1 mg/mL during 24 hours (a). Percentages of GL-261 and RG-2 cell inhibition when these cells are incubated with the various magnetosomes (M-PEI, M-Chi, M-Neri) and coating agents (PEI, Chi, Neri) at a concentration of 1 mg/mL during 72 hours (b). The data presented in this Figure are extracted from Suppl. Fig. 1.

**Suppl. Fig. 3:** (a), Percentage of living RG-2 cells (%) when these cells are brought into contact with 1 mg/mL of MC, M-uncoated, M-Neri, M-Chi, and M-PEI, and either maintained at 37°C during 30 minutes without AMF treatment, black columns: 37°C (-AMF), or exposed during 30 minutes to an AMF of frequency 198 kHz and strength varied between 34 and 47 mT to maintain temperature at between 43°C and 46°C during 30 minutes, red columns: 45°C (+AMF). (b), Spatial temperature distribution, measured with an infra-red camera, of RG-2 cells brought into contact with 1mg/mL of MC, M-uncoated, M-Neri, M-Chi or M-PEI and exposed during 30 minutes to the same AMF as in (a). (c), Quantity of iron coming from magnetosomes, which is internalized in each RG-2 cell when RG-2 cells are brought into contact with 1 mg/mL of MC, M-uncoated, M-Neri, M-Chi, or M-PEI, and exposed during 30 minutes to the same AMF as in (a).

**Suppl. Fig. 4:** variation of the absorbance, measured at 480 nm, of a suspension containing 1mg/mL in iron of uncoated and coated magnetosome minerals in (a) RPMI 20% FBS, (b) DMEM 10% FBS as a function of time, where the absorbance is normalized by the absorbance at the beginning of the measurements.


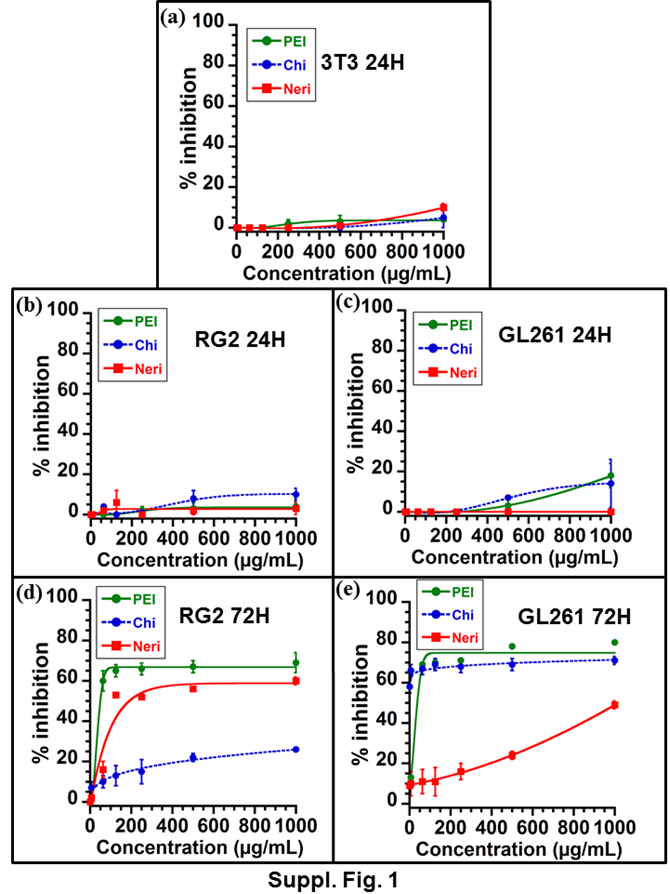

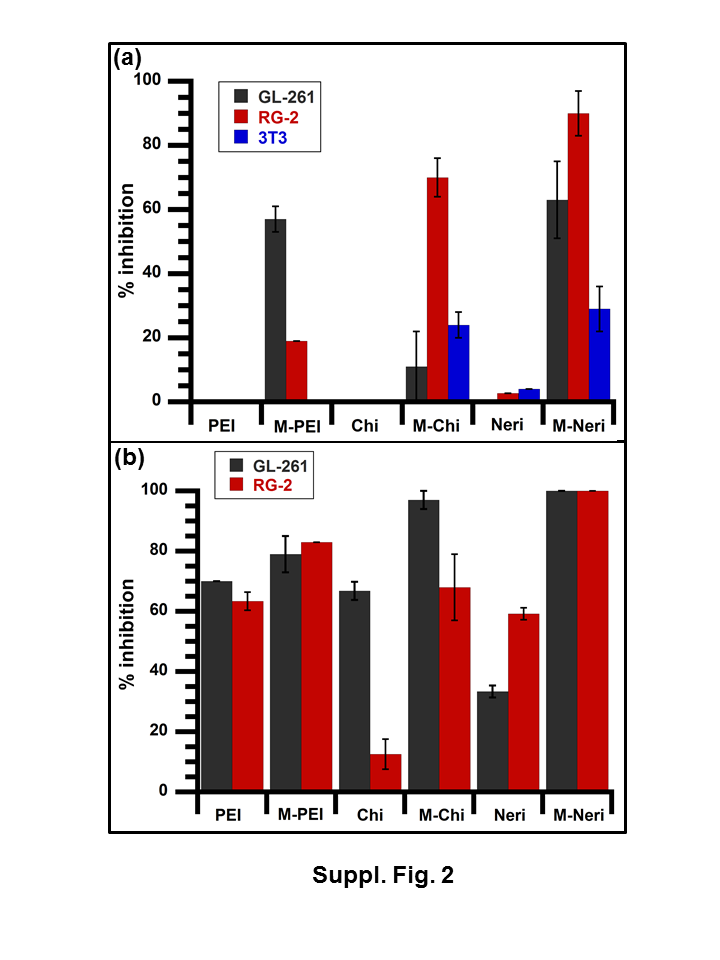

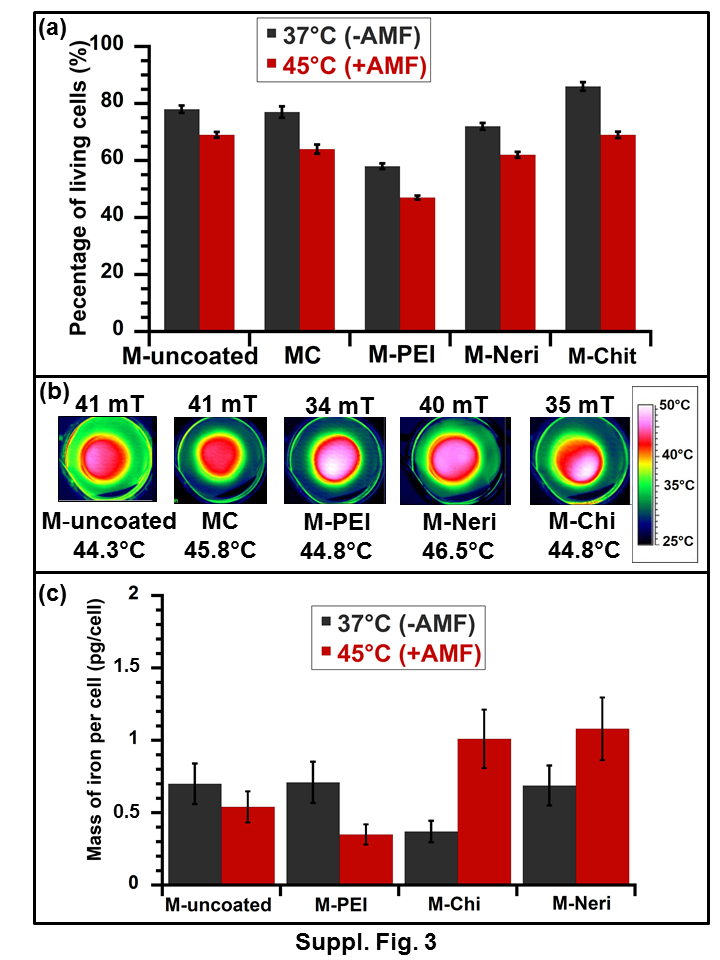

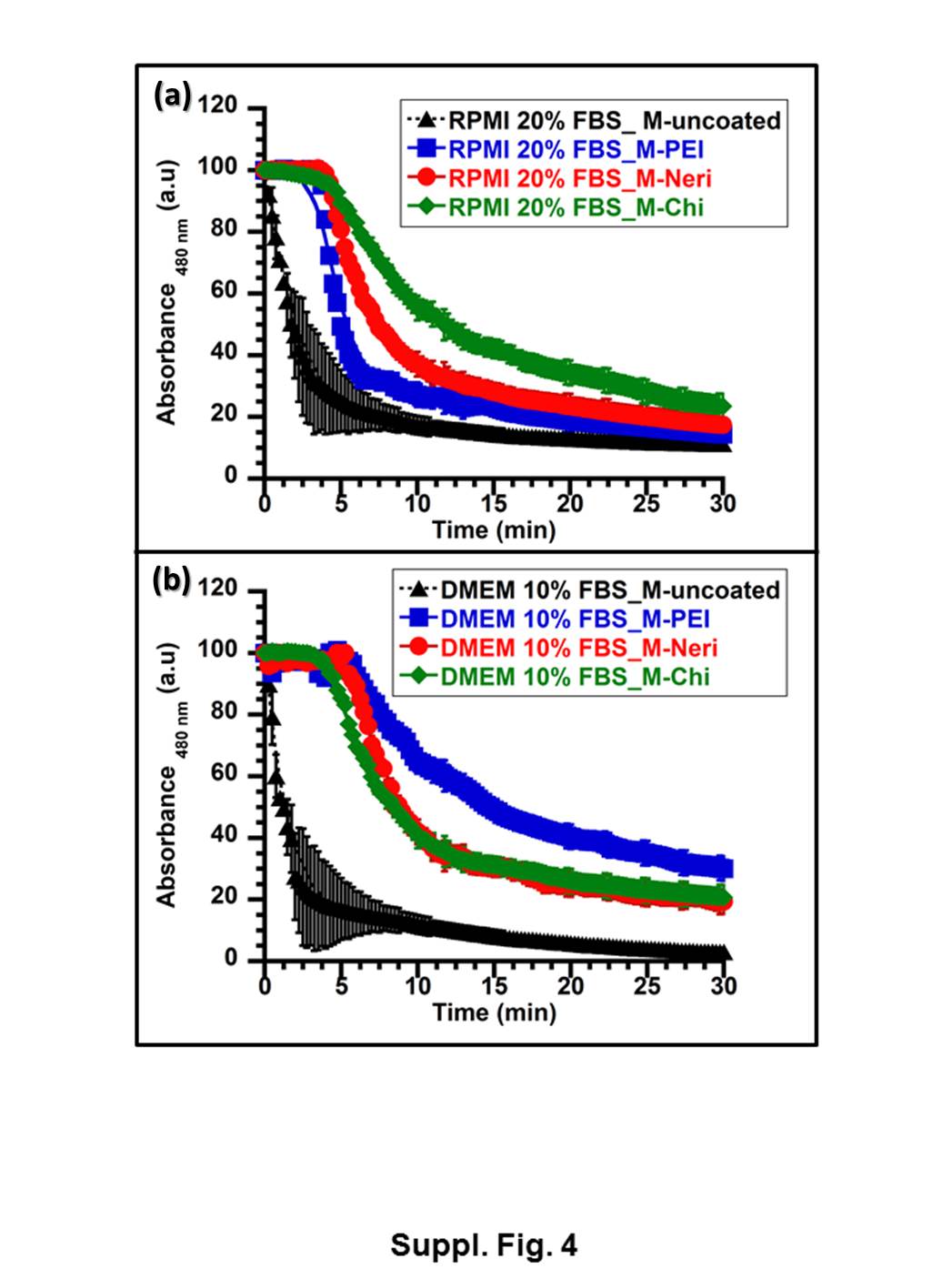

Supplement: Supplementary file 1 — Additional file 1: Figure S1. Percentage of cell inhibition of 3T3 cells brought into contact with the various coating agents for 24 h, (a), of RG-2 cells brought into contact with the various coating agents for 24 h, (b), of GL-261 cells brought into contact with the various coating agents for 24 h, (c), of GL-261 cells brought into contact with the various coating agents for 72 h, (d), and of GL-261 cells brought into contact with the various coating agents for 72 h, (e). Figure S2. Percentages of GL-261, RG-2, and 3T3 cell inhibition when these cells are incubated with the various magnetosomes (M-PEI, M-Chi, M-Neri) and coating agents (PEI, Chi, Neri) at a concentration of 1 mg/mL during 24 h (a). Percentages of GL-261 and RG-2 cell inhibition when these cells are incubated with the various magnetosomes (M-PEI, M-Chi, M-Neri) and coating agents (PEI, Chi, Neri) at a concentration of 1 mg/mL during 72 h (b). The data presented in this Figure are extracted from Additional file 1: Figure S1. Figure S3. (a), Percentage of living RG-2 cells (%) when these cells are brought into contact with 1 mg/mL of MC, M-uncoated, M-Neri, M-Chi, and M-PEI, and either maintained at 37 °C during 30 min without AMF treatment, black columns: 37 °C (−AMF), or exposed during 30 min to an AMF of frequency 198 kHz and strength varied between 34 and 47 mT to maintain temperature at between 43 and 46 °C during 30 min, red columns: 45 °C (+AMF). (b), Spatial temperature distribution, measured with an infra-red camera, of RG-2 cells brought into contact with 1 mg/mL of MC, M-uncoated, M-Neri, M-Chi or M-PEI and exposed during 30 min to the same AMF as in (a). (c), Quantity of iron coming from magnetosomes, which is internalized in each RG-2 cell when RG-2 cells are brought into contact with 1 mg/mL of MC, M-uncoated, M-Neri, M-Chi, or M-PEI, and exposed during 30 min to the same AMF as in (a). Figure S4. Variation of the absorbance, measured at 480 nm, of a suspension containing 1 mg/mL in iro [file 12951_2017_293_MOESM1_ESM.doc]
